# Professor Stuart John Ferguson (29 September 1949–25 April 2024)

**DOI:** 10.1002/2211-5463.13943

**Published:** 2024-12-02

**Authors:** Julie M. Stevens

**Affiliations:** ^1^ Department of Paediatrics University of Oxford UK

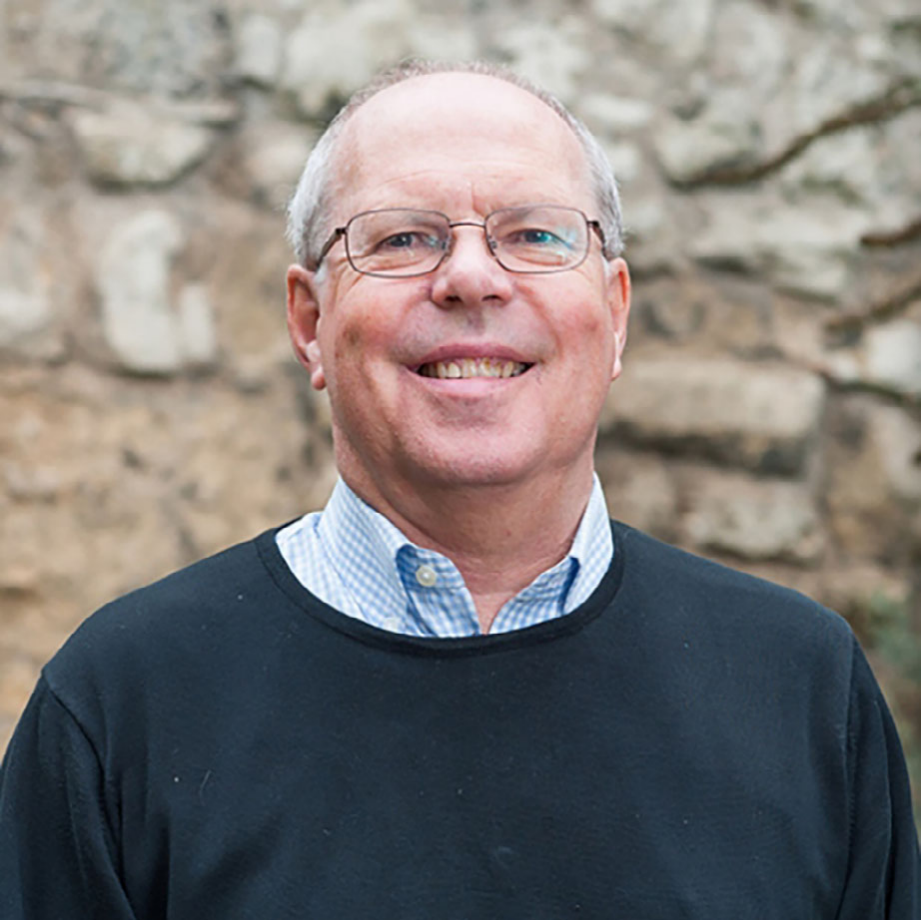

We reflect with great fondness and admiration on the life and work of Stuart Ferguson, following his passing earlier this year at the age of 74. He was an internationally distinguished scientist who leaves a significant legacy. He was a dedicated long‐term member of the Editorial Board of *FEBS Letters* and more recently on the Board of *FEBS Open Bio*, where he was a founding member and a vital contributor to its formation.

Born in the UK, Stuart attended the University of Oxford as an undergraduate in Chemistry, where he was awarded a first‐class degree. He completed his PhD under the supervision of the renowned scientist George Radda, who also passed away recently. Stuart took up a lectureship at the University of Birmingham, where he met and published with Tina George; they later married and had two sons, Robin and George. He returned in 1985 to Oxford to St Edmund Hall, as the William R Miller Tutorial Fellow in Biochemistry. Stuart built a productive multidisciplinary research group in Oxford, with numerous national and international collaborations, and in 1997, he was awarded the title of Professor of Biochemistry. His legacy is marked by exceptional contributions to both research and education in the field of bioenergetics: the very fundamental principles of how energy flows in living systems.

His research discoveries were wide‐ranging and impacted many areas of biology. It happens that Stuart's very first publication (of hundreds) was in *FEBS Letters* in 1972, an NMR study on lysozyme, as was his second paper on one of his favourite protein complexes, ATP synthase.

ATP synthase is central to bioenergetics as it produces ATP, driven by a gradient of ions across membranes. It is a large, multicomponent complex, the mechanism of which took years to elucidate. Stuart conducted a number of key studies on ATP synthase, including a critical early observation using a chemical modification experiment. Modification of only one of the three ATP synthesising components inhibited the entire complex, showing that the three sites were not operating independently. This experiment underpinned the so‐called binding change mechanism, for which Boyer later received a Nobel Prize in Chemistry. With the respect and recognition of the community, Stuart went on to become a thought leader on the subject, notably on P/O ratios, and the intriguing variety of subunits in the c‐rings of the ATPases from different organisms, as detailed structural information became available.

The enzymology of the nitrogen cycle was a significant area of study for Stuart's group and the subject of many collaborations for much of his career. The context for Stuart's interest in the bacterium *Paracoccus denitrificans* was that it is a close relative of the bacterial progenitor of our own mitochondria (a discovery made nearby in Oxford's Botany Department). Mitochondria are known to be remnants of bacteria that colonised our ancestral cells more than a billion years ago by endosymbiosis. Not only is this of great evolutionary interest, but also provided the experimental accessibility of using a bacterial model to understand bioenergetics, preferable to the more cumbersome study of mitochondria as intracellular organelles in more complex organisms. The respiratory adaptability of *Paracoccus* made for fascinating studies and insights: In the absence of oxygen (unlike our own mitochondria which depend on oxygen), *Paracoccus* can use nitrogen oxides as part of their electron transport chains. The process of denitrification is part of the global Nitrogen Cycle, a biogeochemical cycle that is key to life on our planet. Discoveries in this field have informed disciplines from bioremediation to bioenergy production.

The diversity of bacterial bioenergetics proved to be a source of numerous other branches of research, which Stuart pursued with his customary intellectual rigour. The protein cytochrome *c* is a key electron transfer protein in our mitochondria and arises from a post‐translational modification that covalently attaches a single heme molecule to two cysteine side chains in the protein. The work of Stuart and others found a vast array of bacterial cytochromes conferring bioenergetic flexibility, some with many heme molecules, and others with modified hemes, such as the nitrite reductase cytochrome *cd*_1_. The question of how these proteins are formed became a key interest of the Ferguson research group.

Cytochrome *c* biosynthesis describes the process of protein‐mediated covalent attachment of heme to protein and occurs via diverse processes in different cell types. Stuart's work delivered a key range of insights into the proteins in *Escherichia coli* in particular. The Ccm system, a complex set of proteins, attaches the heme molecule, following its transport across the periplasmic membrane, to apocytochromes. Stuart's background in chemistry made for incisive contributions to this area of research.

Disulphide bond formation became another area of interest, because of the involvement of another group of proteins involved in disulphide bond isomerisation between cysteine side chains. The Dsb proteins are essential for oxidative protein folding in the bacterial periplasm, especially proteins that are destined for secretion or targeted to the outer bacterial membrane. Insights into this field have had wider implications for protein stability, biotechnology and importantly in bacterial pathogenicity.

What is most remarkable and unusual about Stuart's contribution is that his commitment to research and discovery was matched by his dedication to teaching and education. His deep expertise led to him coauthoring, with David G. Nicholls, the seminal textbook in the field, named simply *Bioenergetics*, which has seen four editions and is published globally. His tutorials and lectures in Oxford had legendary status as he taught concepts and critical thinking, and shared with his students his scientific motivation of simple curiosity. Stuart continued to contribute to his Oxford college, St Edmund Hall, where he held various senior roles, until and beyond his retirement.

Stuart was awarded the Keilin Medal of the UK Biochemical Society in 2001 for his contributions to the field of bioenergetics. He authored over 250 scientific papers and supervised dozens of doctoral students. Many of those who worked alongside him have gone on to successful careers in industry and academia, having benefitted from Stuart's wisdom, generous mentorship and the freedom to explore and discover in his laboratory.

The thoughts of our community are with Stuart's family, friends, and colleagues. He is profoundly missed, yet his influence leaves an enduring legacy in the field of bioenergetics and in the lives of all who had the privilege of knowing him.

